# Effect of Yeast Fermentate Supplementation on Intestinal Health and Plasma Biochemistry in Heat-Stressed Pekin Ducks

**DOI:** 10.3390/ani9100790

**Published:** 2019-10-12

**Authors:** Jill R. Nelson, Gregory S. Archer

**Affiliations:** Department of Poultry Science, Texas A&M University, College Station, TX 77843, USA; jnelso15@gmail.com

**Keywords:** yeast fermentation product, heat stress, intestinal health, cytokines, ducks

## Abstract

**Simple Summary:**

Yeast cells and cell wall components have been used to improve intestinal health in a variety of livestock. This study examines the addition of the product of yeast fermentation in feed or drinking water on intestinal morphology and blood biochemical measures in mixed-sex Pekin ducks exposed to heat stress or thermoneutral conditions. This study found that when added to the feed or drinking water, yeast fermentate increased villus length, villus/crypt ratio, and plasma uric acid concentrations. As a result, yeast fermentate may support nutrient absorption and modulate amino acid metabolism in mixed-sex Pekin ducks.

**Abstract:**

One experiment was conducted to determine the effects of supplementing *Saccharomyces cerevisiae*-derived yeast fermentate (Diamond V Mills, Cedar Rapids, IA, USA) in the feed (XPC; 1.25 kg/metric ton feed, days 0–35) or drinking water (AviCare; 160 mL/100 L, days 0–35) on plasma biochemical and immune parameters, as well as ileal histomorphology of mixed-sex Pekin ducks grown to 35 d and exposed to cyclic heat stress (8 h/d) or thermoneutral environment (days 21–35). On the day of hatching, 144 straight run White Pekin ducks were randomly assigned to one of six treatments: stressed control (CS), stressed + XPC (XS), stressed + AviCare (AS), non-stressed control (CN), non-stressed + XPC (XN), and non-stressed + AviCare (AN). On day 33, blood samples were collected from 12 birds/treatment to assess plasma chemistry, packed cell volume, and plasma levels of interleukin (IL)-1α, IL-8, and α_1_-acid glycoprotein (α_1_-AGP). On day 34, ileum sections were collected from 12 birds/treatment to assess goblet cell density, villus length, crypt depth, and villus/crypt ratio from 6 villi per sample. Plasma phosphorus was influenced by diet (*p* < 0.001) and heat–diet interaction (*p* = 0.003), and was higher in XS than XN, and higher in AS than AN. Heat stress increased plasma glutamate dehydrogenase (GLDH) (*p* = 0.008). Uric acid was increased by adding yeast fermentate to the feed or drinking water (*p* = 0.002), but was not influenced by heat (*p* > 0.05). The heat–diet interaction affected plasma IL-1α (*p* = 0.021) and sodium (*p* = 0.046). Heat stress reduced villus length (*p* < 0.001), villus/crypt ratio (*p* < 0.001), and goblet cell density (*p* < 0.001), but did not affect crypt depth (*p* > 0.05). Both XPC and AviCare increased villus length (*p* < 0.001) and villus/crypt ratio (*p* < 0.001), and decreased crypt depth (*p* < 0.001), but did not affect goblet cell density (*p* > 0.05). Although adding yeast fermentate to the feed or drinking water does not appear to alleviate the effects of heat stress on goblet cell density, both routes of administration improved other measures of villus morphology and affected amino acid metabolism.

## 1. Introduction

Heat stress is a common problem encountered in commercial poultry houses, particularly in the summer months. Chronic and acute heat stress can compromise intestinal function and negatively impact nutrient absorption, growth rate, and feed efficiency [1]. This is primarily attributed to inflammation, oxidative stress, and the effects of microbial toxins and stress hormones [1,2]. With gut barrier integrity compromised, pathogenic microbes and other debris from the intestinal tract are more likely to enter circulation and cause systemic infection [1]. All of these factors can increase mortality and decrease yield at slaughter. It is important to investigate the mechanisms of heat stress on intestinal barrier function and to find ways to ameliorate or prevent gut barrier failure.

Intestinal morphology is frequently used as an indicator of gut health. Germ cells reside in the crypt between the base of the villus and the basement membrane, and give rise to epithelial cells, which differentiate as they migrate toward the tip of the villus [3]. Mature epithelial cells are continually exfoliated and regenerated. Disruption of the adhesion proteins which connect the enterocyte to nearby cells and the extracellular matrix causes the cell to separate and begin apoptosis: this particular process of induced cell death is called *anoikis* [1,3]. Anoikis is necessary for the maintenance of healthy epithelial cells that line the gastrointestinal tract; however, intestinal integrity can become compromised when stress, inflammation, and other factors stimulate the apoptosis of both germ cells and mature epithelial cells [3]. For example, microbial toxins have been shown to disturb cell proliferation, resulting in reduced crypt depth and villus length [4].

Goblet cells, which also originate from germ cells in the crypt, are distributed along the length of the villus and produce water-soluble glycoproteins that make up the mucus in the intestinal lumen [5]. This acts as a physical barrier against microbes and debris and offers protection against physical damage to the epithelium [4]. Mucus secretion is thought to be stimulated by histamine, acetylcholine, and prostaglandin E2, and is further regulated by interleukin (IL)-10 [5]. However, goblet cell density and mucus secretion can be negatively affected during heat stress and gut barrier failure [6,7]. A number of cytokines have also been used to measure intestinal health and inflammatory processes [7]. Stress hormones, IL-1β, and reactive oxygen species stimulate the transcription of IL-8, which directs immune cells to the site of inflammation [8,9,10]. Alpha-1-acid glycoprotein (α_1_-AGP), which is partially regulated by IL-1β, is a major positive acute phase protein which binds and transports substances of either endogenous or exogenous origin and modulates the immune response [11,12]. Concentrations of α_1_-AGP have been shown to increase during gut barrier failure [7].

Dietary supplementation of whole yeast cells and yeast cell wall components have been tested in poultry. Mannan oligosaccharides and β-glucan, two such components, have been shown to increase the density of certain goblet cell types, as well as villus length [13]. The metabolites contained in the *Saccharomyces cerevisiae*-derived yeast fermentation product Original XPC and its liquid equivalent, AviCare, have demonstrated the ability to reduce measures of stress in broilers during cyclic heat stress [14] and rearing stress [15]. Supplementation of XPC has also been shown to mitigate enteric lesions in ducks infected with Duck Virus Enteritis [16] and intestinal damage in laying hens infected with *Eimeria maxima* [17]. Dairy calves fed XPC during *Salmonella* infection also exhibited greater weight gain and more developed rumens [18]. While there is abundant research available on the effects of whole yeast cells or cell wall components on intestinal health, little exists on the effects of metabolites produced by yeast fermentation. The purpose of this research is to compare measures of intestinal health and plasma biochemistry of mixed-sex White Pekin ducks exposed to either cyclic heat stress or thermoneutral conditions when supplemented with XPC in the feed or AviCare in the drinking water.

## 2. Materials and Methods

### 2.1. General Husbandry

All procedures were carried out in accordance with the guidelines established by Texas A&M Institutional Animal Care and Use Committee (AUP#2018-0135) and the Guide for the Care and Use of Agricultural Animals in Research and Teaching [19]. Birds were housed at the Texas A&M University Poultry Science Teaching, Research, and Extension Center, and all diets were mixed on site. One trial was conducted in which 144 day-of-hatch, mixed-sex White Pekin ducks were randomly assigned to one of six treatments: stressed control (CS); stressed and supplemented with XPC (Original XPC, Diamond V Mills, Cedar Rapids, IA, USA), day 0–35 (XS); stressed and supplemented with AviCare (Diamond V Mills, Cedar Rapids, IA, USA), day 0–35 (AS); non-stressed control (CN); non-stressed and supplemented with XPC, day 0–35 (XN); and non-stressed and supplemented with AviCare, day 0–35 (AN). Original XPC was added to the feed (1.25 kg/metric ton feed), and AviCare was mixed into the drinking water (160 mL/100 L drinking water), according to the manufacturer’s recommended dose. 

There were 24 birds in each treatment. Stressed and non-stressed treatments were separated into two identical rooms in order to control environmental conditions during the cyclic heat stress period. Pens measured 0.91 × 1.83 m and were lined with 3–5 cm of fresh pine shavings. Each pen was equipped with 1 tube feeder and 1 waterer consisting of an 18.93 L plastic bucket with four nipples on the bottom. Birds in each treatment were separated into two pens, with 12 birds/pen. Pens were arranged in a repeating pattern of Control–XPC–AviCare in each room, so that pens from a given treatment were not placed next to each other. Birds were fed a crumbled starter diet on days 0–14 and a pelleted grower diet on days 15–35. The ingredient composition of both diets are shown in Table 1, and nutrient information for both diets is presented in Table 2. Birds had *ad libitum* access to feed and water for the duration of the trial, and feeders and waterers were raised as birds grew. Water in the AviCare pens (AS and AN) was emptied and then refilled from a stock solution daily; all other waterers were refilled as needed.

Room temperature was maintained at 35 °C for 3 d prior to the start of the trial, then reduced to 31 °C for the first 3 d of the trial, and further reduced 2 °C every other day for the next week. Due to high external environmental temperatures, large fans were placed in each room on day 10 to assist with the circulation of cool air from the cooling pad. Birds were provided with 24 h of light for the first 3 d, and 16 h of light (06:00 a.m. to 22:00 p.m.) followed by 8 h of darkness for the remainder of the trial. Starting on day 21 until day 35, birds in the stressed treatments (CS, XS, and AS) were exposed to cyclic heat stress from 08:00 a.m. to 16:00 p.m. daily (8 h/d). This was induced by turning off the cooling pad and the large interior fan circulating cool air, and increasing the heater temperature to achieve a litter temperature between 31–34 °C. Three small vertical-facing fans were evenly spaced on the floor in the heat-stressed room in order to circulate air upwards and mitigate ammonia buildup at the bird level. Birds in the thermoneutral treatments were maintained at a litter temperature between 23–26 °C. At the end of the heat stress period, the cooling pad and large horizontal-facing fan were turned back on to circulate cooler air throughout the room.

### 2.2. Blood Sampling

On day 33, 12 birds from each treatment were chosen at random, and 1–2 mL of blood was collected via the brachial vein and separated between a clot activator serum separation vacutainer (367981, BD Medical, Franklin Lakes, NJ, USA) and a heparin and lithium gel separation vacutainer (367884, BD Medical, Franklin Lakes, NJ, USA). One heparinized hematocrit capillary tube (505, Chase Scientific Glass, Inc., Rockwood, TN, USA) was used to collect whole blood from the heparin vacutainer for each bird, then spun down using a Haematocrit 200 centrifuge (1801, Hettich Group, Kirchlengern, Germany) at 13,000 RPM for 2 min. Tubes were then used to measure packed cell volume (PCV) as a percentage of the total sample in the capillary tube. Vacutainers containing whole blood were inverted 2–3 times and stored in an ice bath until remaining blood samples were collected. Heparin vacutainers were centrifuged (Centrifuge 5804, Eppendorf, Hamburg, Germany) at 4000 RPM for 15 min; plasma was then poured off into a microcentrifuge tube and stored at −20 °C until analysis. Commercially available ELISA kits were used to measure plasma levels of IL-1α (Ch1767, Advanced BioChemicals, Lawrenceville, GA, USA), IL-8 (Ch1234, Advanced BioChemicals, Lawrenceville, GA, USA), and α_1_-AGP (GWB-374Z11, GenWay Biotech, Inc., San Diego, CA, USA). Plasma concentrations of IL-1α, IL-8, and α_1_-AGP were determined by measuring absorbance at 450 nm (Tecan Sunrise, Tecan Trading AG, Switzerland). Samples were assayed in duplicate, and the average of the duplicates was used in statistical analysis. Serum vacutainers were stored horizontally at 4 °C for 3 h until clotting was achieved, and were then centrifuged (Centrifuge 5804, Eppendorf, Hamburg, Germany) at 4000 RPM for 15 min. After centrifugation, 0.5 mL of serum was transferred to a microcentrifuge tube and sent to Texas A&M Veterinary Medical Diagnostic Laboratory (TVMDL) for analysis of plasma chemistry.

### 2.3. Ileum Sampling and Histomorphology Measurements

On day 34, twelve birds were randomly selected from each treatment, and a 1 cm-long section of ileum was collected from the halfway point between the ileocecal junction and Meckel’s diverticulum. Samples were rinsed with phosphate buffered saline and stored in 30 mL of 10% neutral buffered formalin at room temperature. Samples were sent to Histo-Scientific Research Laboratories (Mt. Jackson, VA, USA) to be processed and stained with Periodic Acid-Schiff (PAS) in combination with Alcian Blue (AB). The mounted and stained ileum sections were photographed at 4x magnification, using a Nikon Eclipse Ci-L microscope (Nikon Corporation, Tokyo, Japan). The accompanying Elements software program was used to obtain villus length, crypt depth, villus/crypt ratio, and goblet cell density per 100 µm from six villi per sample.

### 2.4. Statistical Analysis

Plasma biochemical parameters were analyzed using the General Linear Model in Minitab 17.1.0 to determine the main effects of heat (heat stress, no heat stress), diet (control, XPC, AviCare), and heat–diet interaction. Histomorphological data did not meet assumptions for ANOVA; the main effects of heat and diet, and the heat–diet interaction were analyzed using Kruskal–Wallis, followed by pairwise comparisons using the Dwass–Steel–Critchlow–Fligner method [20] for the main effects of heat and diet. A significant difference was defined as *p* < 0.05.

## 3. Results

### 3.1. Plasma Biochemical Parameters and Cytokines

Data for day 33 blood measurements are shown in Table 3 and Table 4. There was a main effect of diet (*p* < 0.001) on plasma phosphorus: control birds had the highest, XPC-supplemented birds had the next highest, and AviCare-supplemented birds had the lowest levels. There was also an interaction effect (*p* = 0.003): AS had higher plasma phosphorus than AN, and XS was higher than XN. There was a trend (*p* = 0.059) toward higher plasma alkaline phosphatase (ALP) in non-heat-stressed birds compared to heat-stressed birds. Temperature also affected plasma glutamate dehydrogenase (GLDH) (*p* = 0.008), which was higher in heat-stressed birds. Diet affected plasma uric acid (*p* = 0.002): both XPC- and AviCare-supplemented birds had higher uric acid than the control, but did not differ from each other. There was an interaction effect on plasma sodium (*p* = 0.046), which was higher in AN and XS compared to CS. An interaction effect was also observed for plasma IL-1α (*p* = 0.021), which showed that levels were highest in CN and lowest in AN.

Neither main effects (*p* > 0.05) nor an interaction effect (*p* > 0.05) was observed for the following plasma biochemical parameters: packed cell volume (PCV), total protein, calcium, glucose, alkaline phosphatase (ALP), creatinine kinase (CK), aspartate aminotransferase (AST), cholesterol, potassium, chloride, Na/K ratio, IL-8, and α_1_-AGP.

### 3.2. Ileum Histomorphology

Data for day 34 ileal measurements are shown in Table 5. Villus length was affected by heat (*p* ≤ 0.001) and diet (*p* ≤ 0.001): heat stressed birds had shorter villi, and both XPC and AviCare had longer villi compared to the control, but were not different from each other. Crypt depth was affected by diet (*p* ≤ 0.001): birds supplemented with XPC or AviCare had lower crypt depth compared to the control, but did not differ from each other. Both heat (*p* ≤ 0.001) and diet (*p* ≤ 0.001) affected the villus–crypt ratio as well. The villus/crypt ratio was lower in heat-stressed birds as well as those fed the control diet. Birds fed XPC or AviCare did not differ from each other in villus/crypt ratio. Heat affected goblet cell density (*p* ≤ 0.001), which was lower in heat-stressed birds.

## 4. Discussion

Glutamate dehydrogenase (GLDH) is an enzyme responsible for catalyzing the reversible conversion of glutamate to α-ketoglutarate, which results in the production of ammonium cations [21]. It is primarily produced by hepatocytes, as well as in the kidneys and cardiac muscle, and its concentration in the blood increases as a result of damage to the liver caused by inflammatory or disease processes [22]. This experiment showed that heat-stressed ducks had higher plasma GLDH concentrations, which has also been demonstrated in heat-stressed parrots [23]. Phosphorus plays a significant role in energy metabolism and bone deposition [24]. Heat stress has been shown to reduce plasma phosphorus in ducks [25]. On the other hand, dietary phosphorus depletion has been shown to reduce GLDH activity [26]. This is because GLDH requires the coenzyme nicotinamide adenine dinucleotide phosphate (NADP) [27]. Heat-stressed birds supplemented with either XPC or AviCare had higher plasma phosphorus than their non-stressed counterparts. In addition, plasma GLDH activity was higher in heat-stressed birds. There appears to be an interaction between plasma phosphorus levels in supplemented treatments and GLDH activity in heat-stressed birds. Dietary supplementation of yeast fermentate may have altered available phosphorus, and as a result, amino acid metabolism. This could explain why plasma levels of uric acid were higher in birds fed XPC or AviCare, although heat stress alone did not affect uric acid concentrations. However, because there was no interaction effect for GLDH, further research is needed to clarify whether yeast fermentate supplementation or heat stress ultimately affected amino acid metabolism.

While uric acid is a waste product of protein metabolism, it also serves to scavenge superoxide radicals. One study found that heat stress altered enzyme activities of catalase and superoxide dismutase, both of which scavenge free radicals produced by oxidative metabolism; however, the study did not assess uric acid concentrations [28]. Uric acid was higher in both XPC and AviCare-supplemented birds, suggesting that adding yeast fermentate to either the feed or the drinking water has similar effects on amino acid metabolism. Yeast fermentate has been shown to improve antioxidant capacity in humans [29]. The increased production of uric acid in ducks supplemented with yeast fermentate may contribute to reduced oxidative damage to intestinal villi. For example, villus length and villus/crypt ratio were higher in supplemented birds compared to the control, and lower in heat-stressed birds. This implies some interaction of heat and diet, although unequal variances prevented the analysis of interactions among histomorphological parameters. However, goblet cell density was reduced in heat-stressed birds and was unaffected by diet. Further research investigating the activity of plasma antioxidative enzymes may clarify any potential relationship between diet-mediated changes in uric acid production and the effects of oxidative stress on ileal histomorphology.

Pekin ducks are thought to be more sensitive to heat stress than other duck breeds [28]. Among the inflammatory cytokines measured, only IL-1α was shown to be affected, and this was higher in CN than AN. Likewise, plasma sodium levels were lowest in CS and highest in XS and AN. Pairwise comparisons of plasma sodium concentrations between XS and XN and between AS and AN were not significant. Therefore, it is unclear whether differences in IL-1α and sodium levels can be attributed to yeast fermentate supplementation or other environmental factors.

Previous studies have shown differences in plasma phosphorus, ALP enzyme activity, and IL-1β in broilers supplemented with XPC or AviCare and reared under thermoneutral conditions [30]. In this study effects observed in plasma GLDH, phosphorus, and uric acid suggest that dietary supplementation of yeast fermentate primarily affects amino acid metabolism. The identities of the metabolites in XPC and AviCare are not currently known, and knowledge about their mode of action is limited thus far. However, this study shows that dietary supplementation of yeast fermentate in either the feed or drinking water supports villus length, crypt depth, and villus/crypt ratio, and increases plasma uric acid and phosphorus in mixed-sex Pekin ducks.

## 5. Conclusions

Heat stress reduced villus length, villus/crypt ratio, and goblet cell density. However, dietary inclusion of yeast fermentation metabolites in the feed or drinking water increased villus length and villus/crypt ratio and therefore, surface area for nutrient absorption. Yeast fermentate may have also affected amino acid metabolism during heat stress by modulating plasma phosphorus levels. Finally, interactions between heat stress and diet were observed for sodium and IL-1α. Future studies investigating plasma antioxidant capacity may clarify associations between dietary yeast fermentate supplementation and amino acid metabolism, oxidative stress, and ileal histomorphology in heat-stressed ducks.

## Figures and Tables

**Table 1:** Ingredient composition (g/kg) of basal diets for each growing period ^1^.

Ingredient	Starter (days 0–14)	Grower (days 15–35)
Corn	433.66	552.26
Soybean meal	396.95	272.82
Wheat middlings	60.17	60.08
DL-Methionine	3.61	2.71
L-Lysine	0.10	0.80
Soybean oil	59.07	79.04
Limestone	26.68	11.84
Monocalcium phosphate	12.54	13.24
Sodium chloride	4.21	4.21
TAMU Trace Mineral Mix ^2^	0.50	0.50
TAMU Vitamin Mix ^3^	2.51	2.51

^1^ Pellet binder (calcium lignosulfonate) was added to both the starter and grower diets at a rate of 2.72 g/kg feed. ^2^ Trace mineral premix added at this rate yields the following per kilogram: 13.33 g manganese, 13.33 g zinc, 13.33 g iron, 1.56 g copper, 0.09 g iodine, a minimum of 1.39 g calcium and a maximum of 1.93 g calcium. Calcium carbonate was used as a carrier. ^3^ Vitamin premix added at this rate yields the following per kilogram: 36,741.67 IU vitamin A; 12,860 IU vitamin D3; 151.67 IU vitamin E; 435.17 mg choline; 153 mg niacin; 67.33 mg D-pantothenic acid; 23.83 mg pyridoxine; 19.83 mg riboflavin; 9.78 mg thiamin; 5.83 mg folic acid; 1.83 mg biotin; and 0.07 mg vitamin B12.

**Table 2:** Nutrient composition of basal diets for each growing period.

Parameter	Units	Starter (d 0–14)	Grower (d 15–35)
Dry Matter (DM)	%	89.21	89.66
Crude Protein	% of DM	23.43	20.63
Crude Fat	% of DM	8.17	10.67
Acid Detergent Fiber	% of DM	4.15	3.46
Ash	% of DM	7.14	5.13
Sulfur	% of DM	0.31	0.30
Phosphorus	% of DM	0.77	0.80
Potassium	% of DM	1.32	1.19
Magnesium	% of DM	0.20	0.20
Calcium	% of DM	1.60	1.16
Sodium	% of DM	0.22	0.20
Iron	ppm (DM)	345.25	339.06
Manganese	ppm (DM)	145.72	105.73
Copper	ppm (DM)	22.31	21.86
Zinc	ppm (DM)	103.69	94.69
Digestible Energy	kcal/kg DM	3831.13	4082.42
Metabolizable Energy	kcal/kg DM	3509.81	3738.12

**Table 3:** Blood plasma chemistry and cytokine levels in straight run White Pekin ducks after 33 days of growth ^1^.

	Packed Cell Volume	Total Protein	Calcium	Phosphorus	Glucose	Alkaline Phosphatase	Creatinine Kinase	Aspartate Aminotransferase	Glutamate Dehydrogenase
Units	%	g/dL	mg/dL	mg/dL	mg/dL	U/L	U/L	U/L	U/L
CS	33.42	3.00	9.98	7.98 ^abc^	199.75	813.33	940.42	17.33	3.27
XS	32.42	3.00	10.03	8.18 ^ab^	204.17	914.67	711.58	17.08	3.17
AS	34.00	3.00	9.93	7.77 ^bc^	204.08	896.08	964.50	16.83	3.83
CN	30.00	3.01	9.72	8.43 ^a^	206.67	870.42	817.83	14.08	2.75
XN	32.25	3.00	10.02	7.56 ^cd^	202.25	1010.92	1028.17	15.75	2.66
AN	32.58	3.02	9.81	7.20 ^d^	205.00	826.10	929.40	16.40	2.50
Pooled Standard Error of the Mean	1.05	0.01	0.17	0.16	3.61	52.47	114.68	1.39	0.34
Main Effect Heat									
Heat	33.28	3.00	9.98	7.98	202.67	874.69	872.17	17.08	3.43 ^a^
No Heat	31.66	3.01	9.85	7.76	204.62	924.88	924.88	15.35	2.64 ^b^
Main Effect Diet									
Control	31.78	3.00	9.85	8.21 ^a^	203.21	879.13	879.13	15.71	3.00
XPC	32.33	3.00	10.03	7.87 ^b^	203.21	869.88	869.88	16.42	2.91
AviCare	33.29	3.01	9.87	7.51 ^c^	204.50	948.55	948.55	16.64	3.23
*p*-Value Heat	0.063	0.143	0.364	0.087	0.507	0.531	0.610	0.147	0.008
*p*-Value Diet	0.342	0.448	0.528	≤0.001	0.916	0.059	0.807	0.791	0.768
*p*-Value Heat–Diet	0.325	0.448	0.778	0.003	0.454	0.292	0.184	0.591	0.418

^a,b,c^ Values with different letters within a row indicate a significant difference at *p* < 0.05. ^1^ CS: Heat-stressed 21–35 d, no supplementation; XS: Heat-stressed 21–35 d and supplemented with XPC (1.25 kg/metric ton feed, 0–35 d); AS: Heat-stressed 21–35 d and supplemented with AviCare (160 mL/100 L drinking water, 0–35 d); CN: Thermoneutral environment 0–35 d, no supplementation; XN: Thermoneutral environment 0–35 d and supplemented with XPC (1.25 kg/metric ton feed, day 0–35); AN: Thermoneutral environment 0–35 d and supplemented with AviCare (160 mL/100 L drinking water, day 0–35). Means represent the average of duplicate assays performed on 12 samples per treatment.

**Table 4:** Blood plasma chemistry and cytokine levels in straight run White Pekin ducks after 33 d of growth ^1^.

	Uric acid	Cholesterol	Sodium	Potassium	Chloride	Na/K ratio	Interleukin-1α	Interleukin-8	α_1_-acid glycoprotein
Units	mg/dL	mg/dL	mEq/L	mEq/L	mEq/L		pg/mL	pg/mL	ng/mL
CS	3.06	148.00	149.89 ^b^	5.09	106.22	29.83	301.15 ^ab^	460.94	3801.79
XS	3.78	155.42	157.67 ^a^	5.12	110.56	31.12	406.94 ^ab^	444.90	3773.30
AS	3.57	150.36	153.82 ^ab^	5.05	109.36	31.06	427.23 ^ab^	499.51	3815.85
CN	2.92	138.64	154.80 ^ab^	5.05	110.80	30.96	446.83 ^a^	405.52	3855.39
XN	4.47	139.09	153.64 ^ab^	5.21	108.73	29.95	333.40 ^ab^	450.79	3781.48
AN	3.69	146.50	155.38 ^a^	4.99	110.75	31.65	282.09 ^b^	492.04	3743.36
Pooled Standard Error of the Mean	0.30	7.39	1.75	0.21	1.37	1.24	121.74	31.77	41.12
Main Effect Heat									
Heat	3.47	151.29	153.79	5.08	108.76	30.70	378.44	468.45	3796.98
No Heat	3.67	141.25	154.52	5.09	110.00	30.77	354.11	449.45	3793.41
Main Effect Diet									
Control	2.99 ^b^	143.52	152.47	5.07	108.63	30.42	373.99	433.23	3828.59
XPC	4.11 ^a^	147.61	155.45	5.17	109.55	30.48	370.17	447.85	3777.39
AviCare	3.62 ^a^	148.52	154.47	5.02	109.95	31.31	354.66	495.78	3779.61
*p*-value Heat	0.371	0.138	0.578	0.985	0.224	0.858	0.576	0.791	0.923
*p*-value Diet	0.002	0.800	0.172	0.783	0.516	0.711	0.928	0.688	0.446
*p*-value Heat*Diet	0.377	0.742	0.046	0.935	0.073	0.623	0.021	0.889	0.375

^a,b^ Values with different letters within a row indicate a significant difference at *p* < 0.05. ^1^ CS: Heat-stressed 21–35 d, no supplementation; XS: Heat-stressed 21–35 d and supplemented with XPC (1.25 kg/metric ton feed, 0–35 d); AS: Heat-stressed 21–35 d and supplemented with AviCare (160 mL/100 L drinking water, 0–35 d); CN: Thermoneutral environment 0–35 d, no supplementation; XN: Thermoneutral environment 0–35 d and supplemented with XPC (1.25 kg/metric ton feed, day 0–35); AN: Thermoneutral environment 0–35 d and supplemented with AviCare (160 mL/100 L drinking water, day 0–35). Means represent the average of duplicate assays performed on 12 samples per treatment.

**Table 5:** Average ileal measurements in straight run white Pekin ducks after 34 d of growth ^1^.

	Villus Length	Crypt Depth	Villus/Crypt Ratio	Goblet Cell Density
Units	μm	μm		#/100 μm
CS	524.57	168.78	3.36	13.02
XS	529.66	151.04	3.85	12.94
AS	519.00	222.29	3.31	12.44
CN	431.13	212.59	2.91	15.9
XN	636.24	169.14	4.55	15.85
AN	653.54	143.69	4.95	15.40
Pooled Standard Error of the Mean	14.13	10.59	0.20	0.72
Main Effect Heat				
Heat	524.41 ^a^	180.70	3.50 ^a^	12.80 ^a^
No Heat	573.64 ^b^	175.14	4.14 ^b^	15.72 ^b^
Main Effect Diet				
Control	477.85 ^a^	190.68 ^a^	3.13 ^a^	14.46
XPC	582.95 ^b^	160.09 ^b^	4.20 ^b^	14.40
AviCare	586.27 ^b^	182.99 ^b^	4.13 ^b^	13.92
*p*-value Heat	≤0.001	0.10	≤0.001	≤0.001
*p*-value Diet	≤0.001	≤0.001	≤0.001	0.96

^1^ CS: Heat-stressed 21–35 d, no supplementation; XS: Heat-stressed 21–35 d and supplemented with XPC (1.25 kg/metric ton feed, d 0–35); AS: Heat-stressed 21–35 d and supplemented with AviCare (160 mL/100 L drinking water, 0–35 d); CN: Thermoneutral environment 0–35 d, no supplementation; XN: Thermoneutral environment 0–35 d and supplemented with XPC (1.25 kg/metric ton feed, 0–35 d); AN: Thermoneutral environment 0–35 d and supplemented with AviCare (160 mL/100 L drinking water, 0–35 d). Means represent the average of measurements taken from six villi from each of 12 birds per treatment (72 measurements per treatment).
